# Supplementary material for: micronuclAI enables automated quantification of micronuclei for assessment of chromosomal instability
Source: Commun Biol. 2025 Mar 4;8:361. doi: 10.1038/s42003-025-07796-4 (PMC11880189; doi:10.1038/s42003-025-07796-4)
Supplement: Supplementary file 2 — Supplementary Information [file 42003_2025_7796_MOESM2_ESM.pdf]

Supplementary figures:

Supplementary Figure 1: Detailed workflow of the manual quantification and micronuclAI.

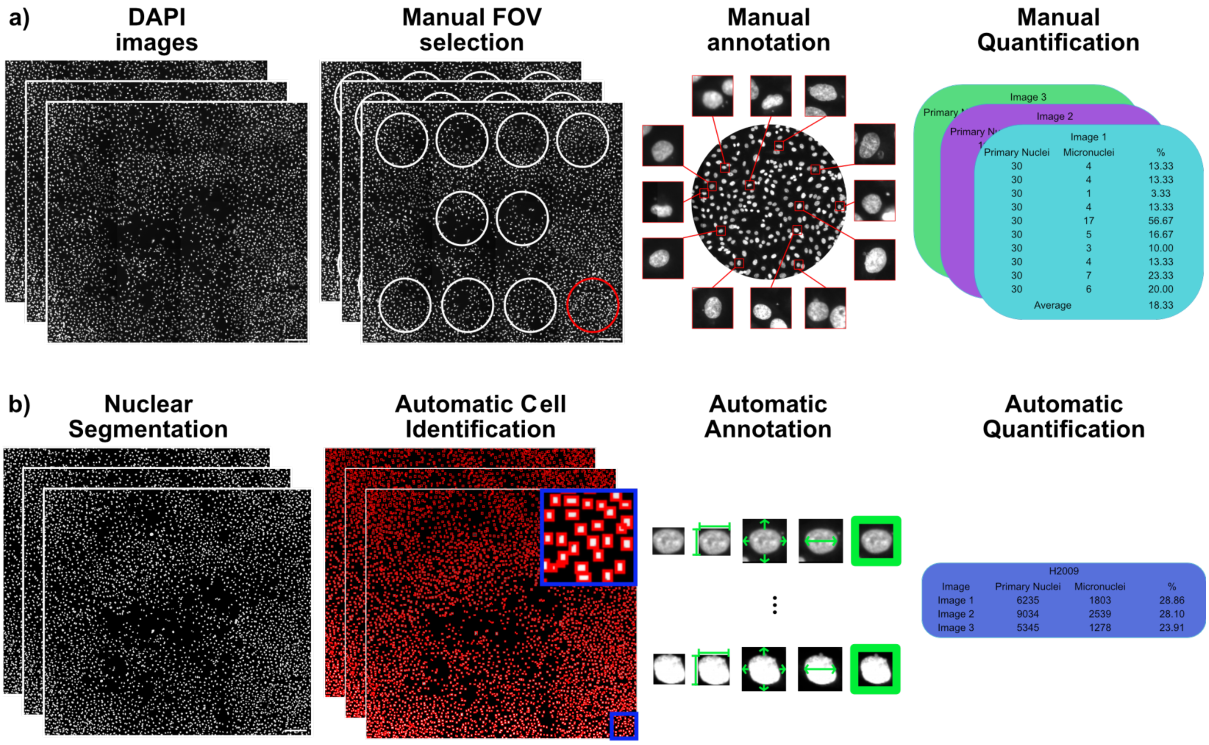

Figure S1: micronuclAI replicates manual quantification workflow. a, manual workflow for micronuclei quantification and **b,** the proposed automatic quantification workflow with micronuclAI. (All scale bars = 100µm)

Supplementary Figure 2: Example of various segmentation methods on CIN predictions.

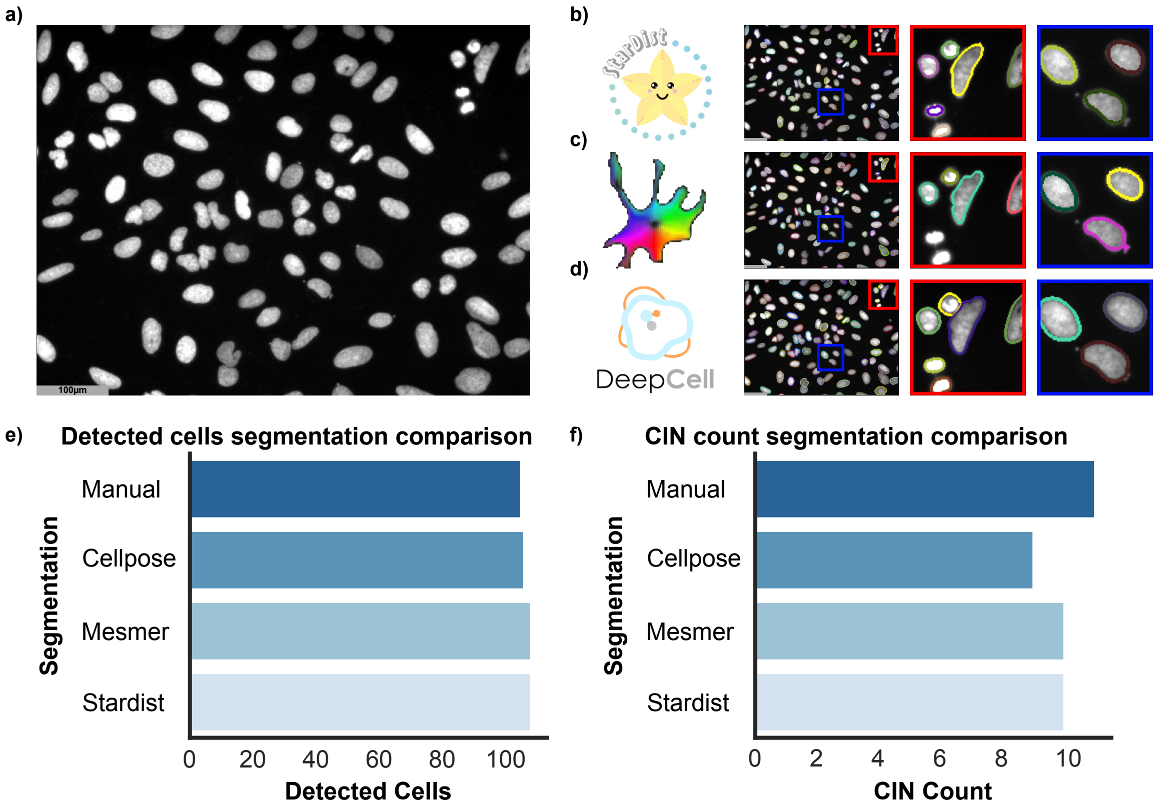

Figure S2: micronuclAi performance is stable across segmentation methods. a, Example of Hoechst-stained image and the segmentation overlays with zoom-in regions for three segmentation methods: **b,** Stardist, **c,** Cellpose, and **d,** DeepCell nuclear segmentation. Comparison of the number of **e,** detected cells and **f,** CIN counts across manual counting and the segmentation methods. (All scale bars = 100µm)

Supplementary Figure 3: Examples of cases where the model does not perform well

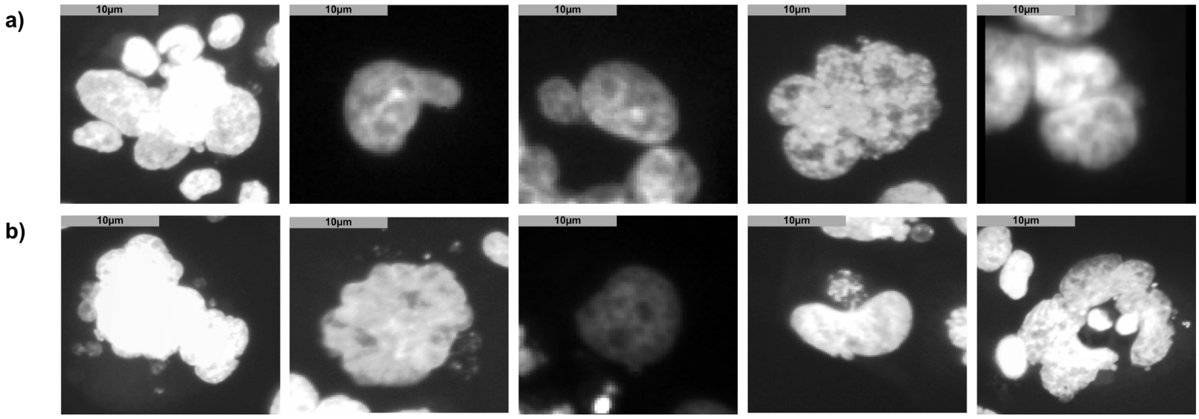

Figure S3: micronuclAI detection challenges in overlapping and overcrowded cell environments. **a**, Overlapping cells growing on top of each other can sometimes appear to contain micronuclei (MN), as their nuclei are often segmented as a single entity. In such cases, micronuclAI typically provides a score of 0 MN; however, in some instances, additional nuclei are misinterpreted as nuclear buds, resulting in a score of 1 MN. **b**, Overcrowding in the field of view (FOV), where nuclei are surrounded by neighboring nuclei or fragments, can lead to misidentification, particularly when adjacent nuclei are undergoing e.g., apoptosis. This disintegration can mimic the appearance of multiple micronuclei, complicating accurate MN identification. (All scale bars = 10μm)

Supplementary Figure 4: Virtual Reality Approach for annotations

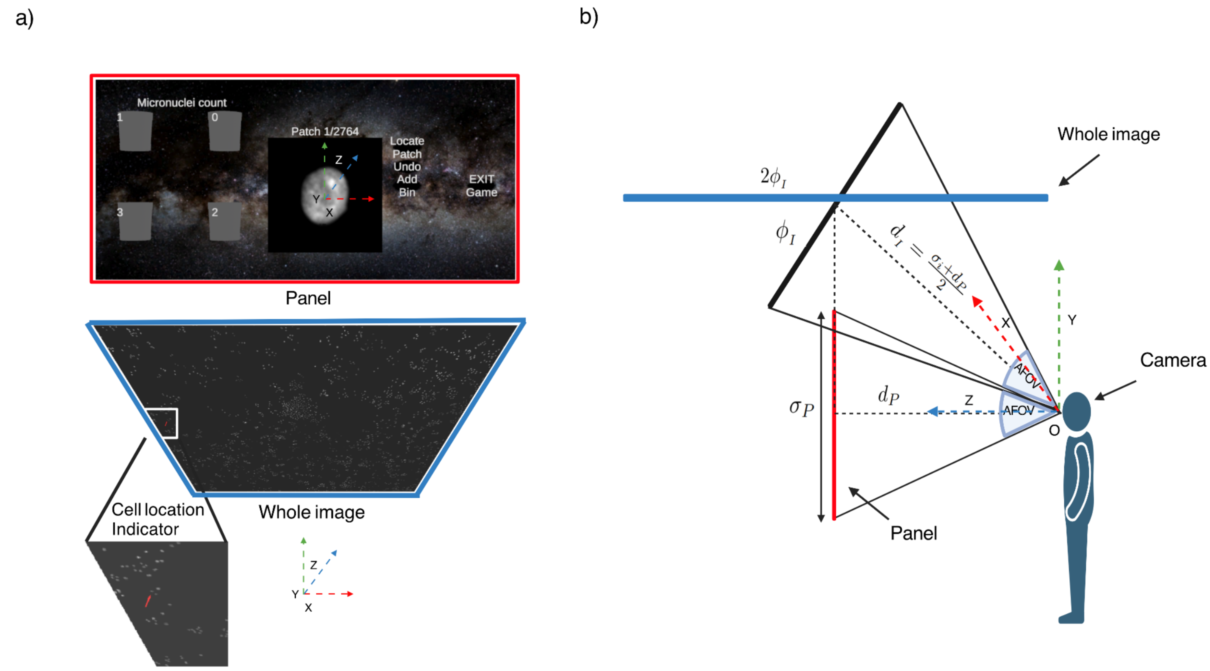

Figure S4: **micronuclAI VR annotation tool. a,** Visualization of the VR work space, where the user classifies single nuclei patches to record their micronuclei count. Selection “buckets” are located on a left side panel and are assigned integers corresponding to the micronuclAI count. The annotator will drag the current single nuclei patch into the corresponding bucket, thus recording the annotation to a TSV file. Additionally, the user can scale patches up or down for improved inspection and have the patch position indicated by an arrow pointing to the bounding box on the whole image. Upon session completion, the TSV file is saved to later be loaded as ground truth matrix for training the deep learning model. **b,** Schematic of the visualization of object arrangement in the VR workspace.

Symbols: δ_p: Panel height; d_P: Working distance related to panel; δ_i: Patch height; ϕ_I: Vertical field of view related to whole image; d_I: Working distance related to whole image; **O**: 3D coordinate space origin; AFOV: Vertical angular field of view

Supplementary Tables:

Supplementary Table 1: List of annotated datasets

Dataset	Cell line	Modifications	No. of Images	No. of Nuclei	Objective	Staining
A375	Malignant Melanoma	control/ Kif2a MCAK/dnMCAK	23	84,286	10	DAPI
H358	Human Non-small cell lung cancer	control/ GFP/ dnMCAK	17	19,954	10	DAPI
Broad BBBC039v1	Human osteosarcoma (u2os)	none	20	1,999	20	Hoechst
KP/KL	Mouse Non-small cell lung cancer	none	12	9,237	10	DAPI
Total			72	115,476		

Supplementary Table 2: Average 10 fold cross validation performance for balanced and unbalanced datasets trained with different model architectures

Downsampling	Base Model	K-Folds	Size	Mean-F1	Mean- RMSE
Balanced	EfficientNet V1 - B0	10	256	**0.9186**	**0.3057**3
Unbalanced	EfficientNet V1 - B0	10	256	0.901	0.34259
Balanced	EfficientNet V1 - B0	10	128	**0.9147**	**0.3178**
Unbalanced	EfficientNet V1 - B0	10	128	0.8954	0.3482
Balanced	EfficientNet V2 - S	10	256	**0.9215**	**0.300**8
Unbalanced	EfficientNet V2 - S	10	256	0.8989	0.3561
Balanced	EfficientNet V2 - S	10	128	**0.9229**	**0.300**8
Unbalanced	EfficientNet V2 - S	10	128	0.8954	0.3483

Supplementary Table 3: Average 10 fold cross validation performance for models with (~10%) and without (Clean) presence of blurry nuclei images.

Downsampling	Data QC	Base Model	K-Folds	Size	F1	RMSE
Balanced	Clean	EfficientNet V2 - S	10	128	**0.9225**	**0.2997**
Unbalanced	Clean	EfficientNet V2 - S	10	128	0.9004	0.3410
Balanced	10%	EfficientNet V2 - S	10	128	**0.9186**	**0.3106**
Unbalanced	10%	EfficientNet V2 - S	10	128	0.8990	0.3425
