# Supplementary material for: micronuclAI enables automated quantification of micronuclei for assessment of chromosomal instability
Source: Commun Biol. 2025 Mar 4;8:361. doi: 10.1038/s42003-025-07796-4 (PMC11880189; doi:10.1038/s42003-025-07796-4)
Supplement: Supplementary file 3 — Description of Additional Supplementary File [file 42003_2025_7796_MOESM3_ESM.pdf]

Description Of Additional Supplementary File

File name: Supplementary Data
Description: numerical source data for figure 4.
